# Myocardial fibrosis: why image, how to image and clinical implications

**DOI:** 10.1136/heartjnl-2019-315560

**Published:** 2019-10-24

**Authors:** Rong Bing, Marc Richard Dweck

**Affiliations:** 1 Centre for Cardiovascular Science, University of Edinburgh, Edinburgh, UK; 2 Centre for Cardiovascular Science, University of Edinburgh, Edinburgh, UK

**Keywords:** myocardial disease, cardiac magnetic resonance (CMR) imaging

**Learning objectives**

- Understand the pathophysiology of myocardial fibrosis.
- Understand the core concepts of cardiovascular MRI for myocardial fibrosis.
- Understand the clinical implications of myocardial fibrosis imaging in common myocardial diseases.

## Introduction

The current era has seen major advances in myocardial imaging. We are now able to assess cardiac anatomy, function, tissue composition and disease activity across a wide range of disease states. Recently, there has been major interest in imaging myocardial fibrosis, predominantly with cardiovascular magnetic resonance (CMR). Fibrosis represents a common response to injury in most cardiomyopathies, but the distribution and pattern of fibrosis differ between pathologies, underscoring its potential role as a diagnostic marker. Furthermore, fibrosis is closely associated with impaired left ventricular function, as well as cardiac arrhythmias; as such, fibrosis imaging also provides powerful prognostic information.

In this review, we discuss the pathophysiology of myocardial fibrosis, review the applications of CMR in the non-invasive detection of myocardial fibrosis and provide an overview of common pathologies in which fibrosis imaging may be of clinical utility.

## Pathophysiology

Our understanding of myocardial function and disease is largely focused on cardiomyocytes. However, the majority of cardiac cells in the adult mammalian heart are non-myocytes. Cardiac fibroblasts are one such cell type that play a crucial role in myocardial disease and healing. These cells are responsible for the production and deposition of extracellular matrix proteins—type 1 collagen being the prototype—which serve as a scaffold for other cellular components and are integral to the structural integrity and function of the myocardium. Fibroblasts respond to cytokines and neurohormal factors, differentiating into activated fibroblasts and smooth muscle-like myofibroblasts that are critical in the healing response of diseased or injured myocardium. These cells exert their effects on the extracellular matrix via regulation of matrix metalloproteinases and fibronectin. Although the primary response to myocardial injury—wound healing, reparative scar formation and remodelling—is important in limiting tissue damage, chronic changes become maladaptive, ultimately impairing cardiac function.

Myocardial fibrosis is a common final pathway in chronic myocardial disease and is the structural correlate of heart failure. It has traditionally been divided into interstitial fibrosis and replacement fibrosis, although more recent histology/CMR data have suggested considerable overlap between these two states.^1^ Diffuse interstitial fibrosis occurs earlier in the course of disease and represents collagen synthesis and deposition by differentiated myofibroblasts in response to a variety of stimuli. Importantly, reactive interstitial fibrosis is reversible. A subgroup of interstitial fibrosis encapsulates infiltrative pathologies that deposit proteins in the interstitium (eg, cardiac amyloidosis). Replacement fibrosis represents collagen deposition that occurs following myocyte apoptosis or necrosis. Replacement fibrosis is irreversible and is of prognostic relevance across a broad spectrum of myocardial diseases. The ability to detect and quantify fibrosis non-invasively—rather than relying on biopsies, which are limited by sampling error and the inability to assess the entire myocardium—is therefore of major clinical interest.

## Imaging techniques

A non-invasive technique to assess myocardial fibrosis requires excellent temporal and spatial resolution in addition to the ability to characterise soft tissue. CMR is therefore the imaging modality of choice. Although CT has also been studied,^2^ it is inferior to CMR for soft-tissue characterisation. We will focus on the most common CMR techniques for fibrosis imaging—late gadolinium enhancement (LGE) and T1 mapping.

### CMR: replacement fibrosis

The detection and quantification of replacement fibrosis using CMR is an established technique and has a larger evidence base than techniques which assess diffuse interstitial fibrosis. It requires the use of gadolinium-based contrast agents (GBCAs) which shorten the T1 of tissues in which they accumulate, providing a high-intensity signal on T1-weighted imaging. These large molecule agents distribute at different rates into healthy and diseased myocardium, partitioning into extracellular space and thus washing out of regions of focal replacement fibrosis at a slower rate than healthy tissue. The contrast in signal provides a visual difference between areas of replacement fibrosis (white) and healthy myocardium (black), although it is insensitive for detecting reactive interstitial fibrosis due to the diffuse nature of this type of fibrosis. Importantly, manual selection of the inversion time to null the myocardium at the time of acquisition is required to achieve this visual contrast, introducing some subjectivity. Late gadolinium enhancement (>7 min after injection, LGE) is the standard parameter and is usually presented as either a dichotomous finding or a percentage of myocardial volume. Both the presence and volume of LGE are almost universally associated with a poorer prognosis in myocardial disease. Different patterns of LGE are associated with different disease states (table 1).

**Table 1:** Typical CMR fibrosis findings in common pathologies

	LGE	T1 mapping
Ischaemic cardiomyopathy	Subendocardial involvement Variable transmural extension Coronary artery territory distribution	Quantitative native T1 may perform similarly to LGE for detecting chronic infarction ECV and native T1 in non-infarcted myocardium appear to be elevated
DCM	Non-ischaemic distribution, often mid-wall/subepicardial	ECV and native T1 may be elevated
Aortic stenosis	Typically non-ischaemic mid-wall distribution May have subendocardial involvement	ECV, iECV and native T1 may be elevated Post-AVR findings vary depending on relative regression of cellular and extracellular constituents of myocardium.
Hypertrophic cardiomyopathy	Patchy non-ischaemic distribution in regions of focal wall thickening, or at the right ventricular insertion points in the septum	ECV and native T1 may be elevated, even in patients without LGE
Myocarditis	At least one focal lesion in non-ischaemic distribution; often inferolateral and subepicardial Used in conjunction with T2 mapping and early gadolinium enhancement for oedema and hyperaemia	Native T1 may offer greater diagnostic accuracy in myocarditis than LGE and traditional Lake Louise criteria Not specific for acute vs chronic myocarditis
Cardiac amyloidosis	Diffuse myocardial uptake Difficult to null images —black blood pool rather than white	ECV and native T1 elevated and may quantify disease burden
Cardiac sarcoidosis	Non-specific appearances Multi-focal, non-ischaemic distribution is suggestive	Native T1 may discriminate sarcoidosis from healthy controls Regresses with anti-inflammatory therapy

AVR, aortic valve replacement; CMR, cardiovascular magnetic resonance; DCM, dilated cardiomyopathy; iECV, indexed extracellular volume; LGE, late gadolinium enhancement.

### CMR: interstitial fibrosis

**Figure 1:**
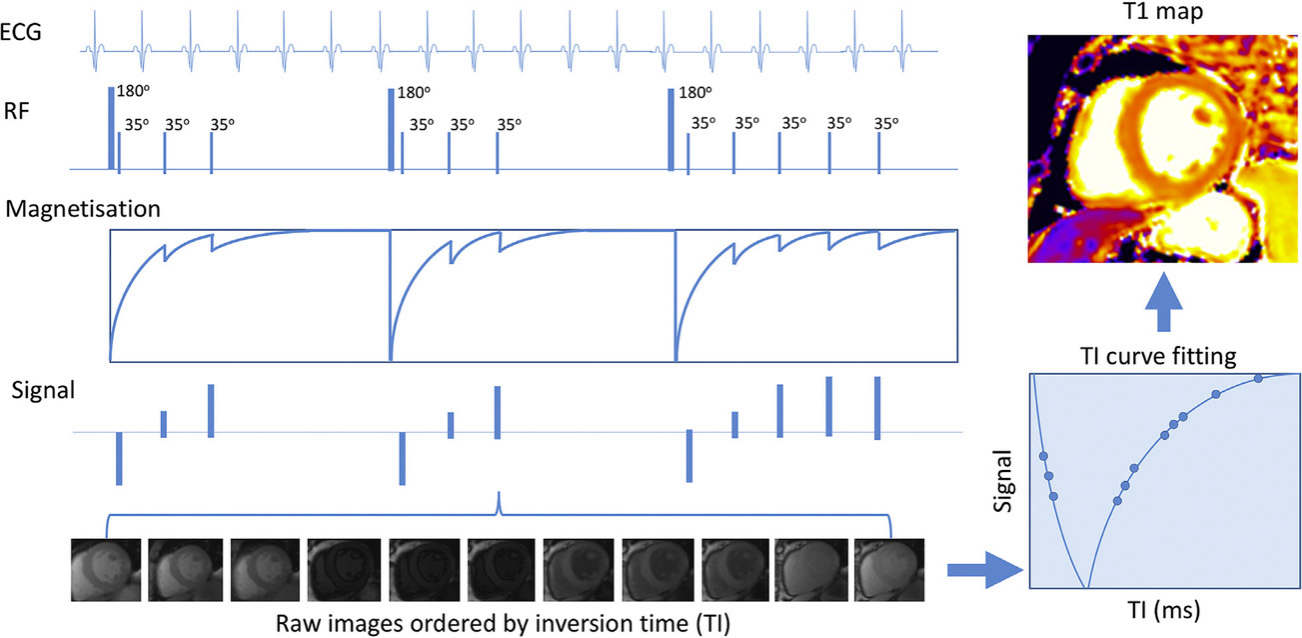
T1 mapping in cardiovascular magnetic resonance modified Look-Locker inversion recovery sequence. A sequence of three inversion recovery experiments are performed with images acquired and ordered according to inversion times. Signals are then used to plot a T1 recovery curve. The T1 value is the time when T1 recovery is 63% complete. T1 values are then used to create a voxel map. Adapted from Everett *et al*.^58^

T1 mapping is the cornerstone of interstitial fibrosis imaging with CMR. There are several different approaches, including native T1, extracellular volume fraction (ECV%) and indexed extracellular volume (iECV). These techniques are comprehensively reviewed elsewhere^3^ and guidelines for their use have been published.^4^ Briefly, T1 mapping interrogates tissue recovery from longitudinal magnetisation (relaxation) following saturation (90 degrees) or inversion (180 degrees) pre-pulses (figure 1). Various protocols are used. Images are acquired at several timepoints during recovery, with T1 times encoded as signal intensities within each voxel. Clinical interpretation is facilitated by applying colour look-up tables for visual assessment; various vendors may supply automated colour maps.

Native T1 values (milliseconds) are measured without GBCAs and reflect the combined intracellular and extracellular compartments. Values increase with a greater burden of fibrosis and are usually measured on a per-segment basis. In contrast, ECV% (percentage) and iECV (mL or mL/m^2^) use GBCAs to target the extracellular space. ECV% represents the extracellular matrix as a proportion of total left ventricular myocardial volume, whereas iECV adjusts for left ventricular myocardial volume (ECV%×left ventricular myocardial volume) and offers a measure of absolute matrix volume.^5 6^

A barrier to the widespread adoption of T1 mapping has been standardisation between vendors and sequences and unclear thresholds for normal values. Recently, extensive research has been conducted as part of the International T1 Multicentre Outcome CMR Study (NCT02407197, NCT03749343) to develop and validate cross-vendor sequences that are transferable, reproducible and easy to acquire. Consequently, T1 mapping is now at the forefront of myocardial imaging research.

Given the distinct differences between reactive interstitial fibrosis and replacement fibrosis, the combination of CMR T1 mapping and LGE offers a comprehensive assessment of myocardial disease that is currently unmatched by any other imaging modality.

## Fibrosis imaging in myocardial diseases

Imaging of myocardial fibrosis using CMR has been studied in a variety of cardiovascular conditions for both diagnosis and prognosis. Guidelines endorsing standardised image acquisition and analysis have been published.^7^ We will discuss some of the more common clinical applications of fibrosis imaging.

### Ischaemic cardiomyopathy and myocardial infarction

**Figure 2:**
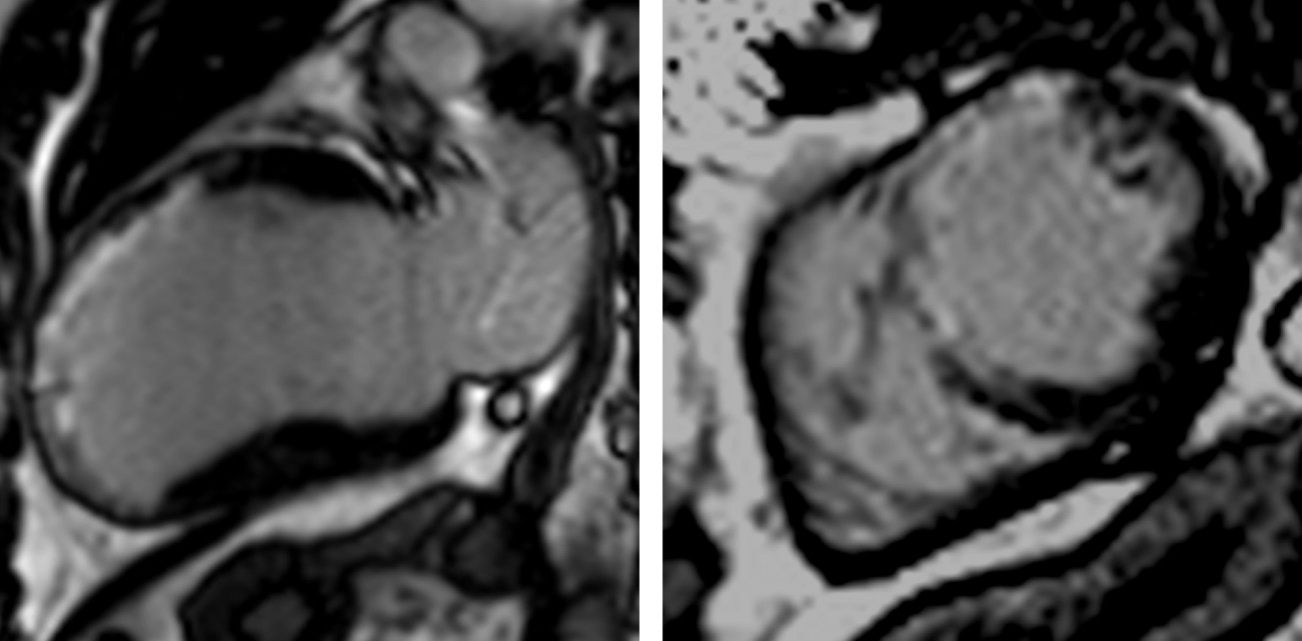
Ischaemic cardiomyopathy extensive anteroseptal myocardial infarction. Two-chamber (left) and short-axis (right) views demonstrate transmural late gadolinium enhancement in the left anterior descending artery territory with associated wall thinning, suggesting no viability.

Myocardial infarction is the prototypical model of myocyte necrosis, apoptosis and collagen deposition with the formation of scar (replacement fibrosis). LGE in myocardial infarction occurs in epicardial coronary artery territories and may be subendocardial or transmural, often accompanied by regional wall motion abnormalities or wall thinning (figure 2). The most common clinical role for myocardial fibrosis imaging in ischaemic heart disease is to establish viability.^8^ Although transmurality of LGE is a continuum, wall motion recovery rarely occurs if >75%. The presence and quantity of LGE is a strong independent predictor of adverse outcomes and mortality.^9–11^

In addition to the prognostic implications of LGE, it is important to appreciate the diagnostic utility of CMR in suspected myocardial infarction. In the current era of high-sensitivity cardiac troponin, there is often diagnostic uncertainty in patients with chest pain, a normal ECG and a small elevation in troponin concentration, particularly when obstructive coronary artery disease is not observed on angiography. In these cases of myocardial injury with non-obstructed coronary arteries, CMR may offer a diagnosis based on the pattern of injury and myocardial dysfunction.^12^ Furthermore, in patients with suspected non-ST elevation myocardial infarction (NSTEMI) who proceed to coronary angiography, the culprit lesion if often unclear. A recent prospective study in 114 patients presenting with their first NSTEMI elegantly demonstrated the ability of LGE on CMR to alter either the diagnosis and/or the designated culprit lesion in nearly half the cohort.^13^

T1 mapping has also been investigated. Studies in patients with myocardial infarction have demonstrated the ability of T1 mapping to quantify infarct size and differentiate reversible and irreversible myocardial injury,^14 15^ while recent research in stable coronary artery disease has shown the prognostic importance of native T1 and ECV in non-infarcted myocardium.^16 17^ However, further studies are now required to determine whether T1 mapping has clinical utility as a dynamic biomarker to guide therapies.

### Dilated cardiomyopathy

**Figure 3:**
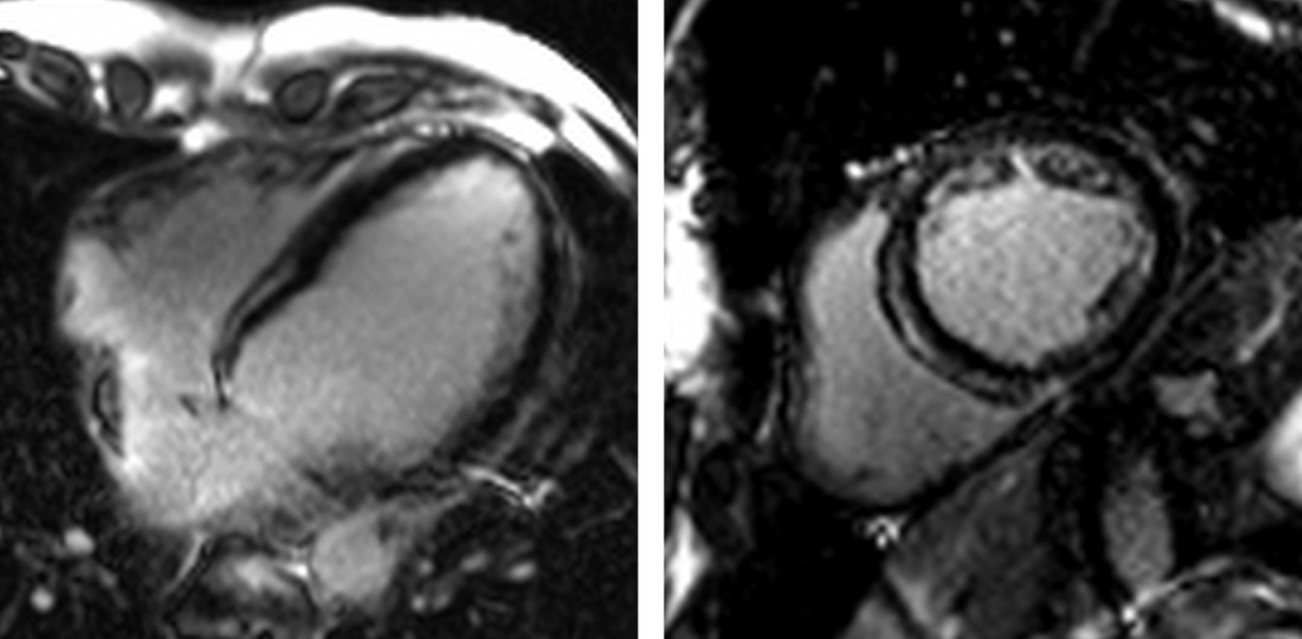
Non-ischaemic dilated cardiomyopathy. Four-chamber (left) and short-axis (right) views demonstrate anteroseptal and inferoseptal late gadolinium enhancement in a typical non-ischaemic (mid-wall) distribution. Note sparing of the subendocardium.

Dilated cardiomyopathy (DCM) is a major cause of cardiovascular morbidity and mortality, with heterogeneous mechanisms governing ventricular dysfunction, arrhythmias and sudden cardiac death. CMR can aid characterisation of the underlying pathology (figure 3). Around a third of patients with DCM demonstrate a non-ischaemic pattern of LGE (mid-wall or subepicardial), which is again a predictor of adverse outcomes, including heart failure, ventricular arrhythmias, sudden cardiac death and all-cause mortality.^11 18 19^ T1 mapping has also been investigated in DCM, providing complementary information to LGE. T1 mapping may discriminate healthy from diseased myocardium, correlating with histology.^20 21^ Most recently, investigators have shown T1 mapping to be a strong predictor of all-cause mortality and heart failure, death or hospitalisation, independent of standard measures of risk such as ejection fraction and functional status.^17 22^

These early data highlight the need for more prospective studies of myocardial fibrosis imaging as a risk stratifying tool in DCM. In light of recent data,^23^ there is currently equivocation regarding the optimal use of implantable cardioverter-defibrillators for primary prevention in DCM. Indeed, many clinicians opt not to proceed with this intervention in patients who meet current guideline-recommended criteria.^24^ Studies such as CMR GUIDE (NCT01918215), a randomised controlled trial allocating patients with mild–moderate left ventricular systolic dysfunction to primary prevention implantable cardioverter-defibrillator or loop recorder based on LGE,^25^ are keenly awaited.

### Aortic stenosis

Aortic stenosis is the best-studied valvular heart disease with regards to myocardial fibrosis imaging. In recent years, there has been a paradigm shift in the assessment and risk stratification of aortic stenosis, with more focus on the myocardium. Current European Society of Cardiology guidelines recommend aortic valve intervention in symptomatic aortic stenosis, reflecting the standard clinical practice in place for many years.^26^ However, there is major uncertainty regarding the timing of intervention in asymptomatic patients. Guidelines suggest a variety of factors that may influence this decision—for example, an ejection fraction <50%, elevated brain natriuretic peptide levels or a peak aortic velocity >5.5 m/s—but these are weak recommendations supported by level C evidence.

**Figure 4:**
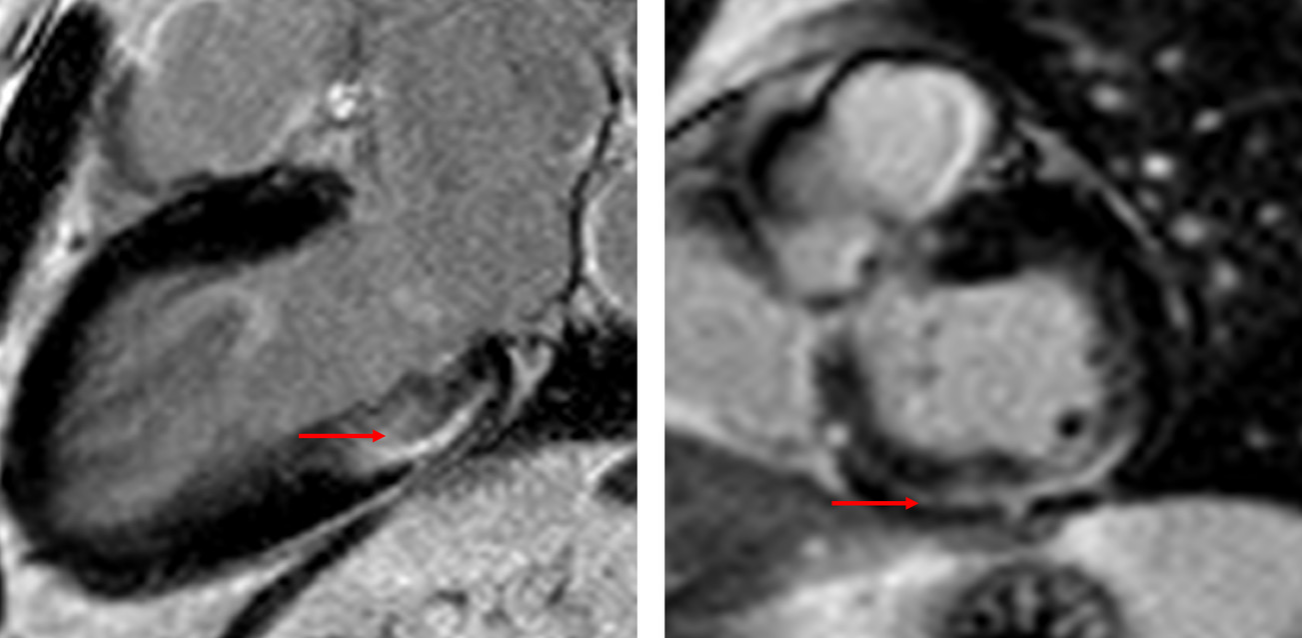
Aortic stenosis myocardial fibrosis in aortic stenosis. There is non-ischaemic late gadolinium enhancement in the basal inferolateral and inferior wall, where the subendocardium is spared (red arrow).

As such, there is a clinical need for more sophisticated risk stratification. CMR fibrosis imaging has the potential to fill this role.^27^ Myocardial fibrosis in aortic stenosis reflects chronically elevated left ventricular afterload which results in a number of pathological changes including cellular hypertrophy, expansion of the extracellular matrix and ischaemia due to supply–demand mismatch (figure 4). Multiple cohorts have demonstrated the presence of both infarct and non-infarct LGE to be independently associated with mortality.^5 28–31^ Furthermore, once established, replacement fibrosis progresses rapidly and does not regress after aortic valve replacement.^6 32^ This is particularly important as there appears to be a “dose-dependent” association between LGE and cardiovascular as well as all-cause mortality.^31^ These observations have formed the basis for current prospective research, including the EVOLVED trial (NCT03094143), which is the first randomised controlled trial to use LGE as a trigger for aortic valve replacement in asymptomatic severe aortic stenosis.^33^

The natural progression of myocardial fibrosis imaging in aortic stenosis is the investigation of diffuse interstitial fibrosis—that is, detecting myocardial disease at an early, reversible stage. This is relevant as interstitial fibrosis can regress after aortic valve replacement.^6^ A number of observational studies have demonstrated correlations between diffuse fibrosis parameters (native T1, ECV% and iECV) and histology; however, data investigating the associations between these markers and clinical outcomes are limited at present.^5 34 35^

### Hypertrophic cardiomyopathy

**Figure 5:**
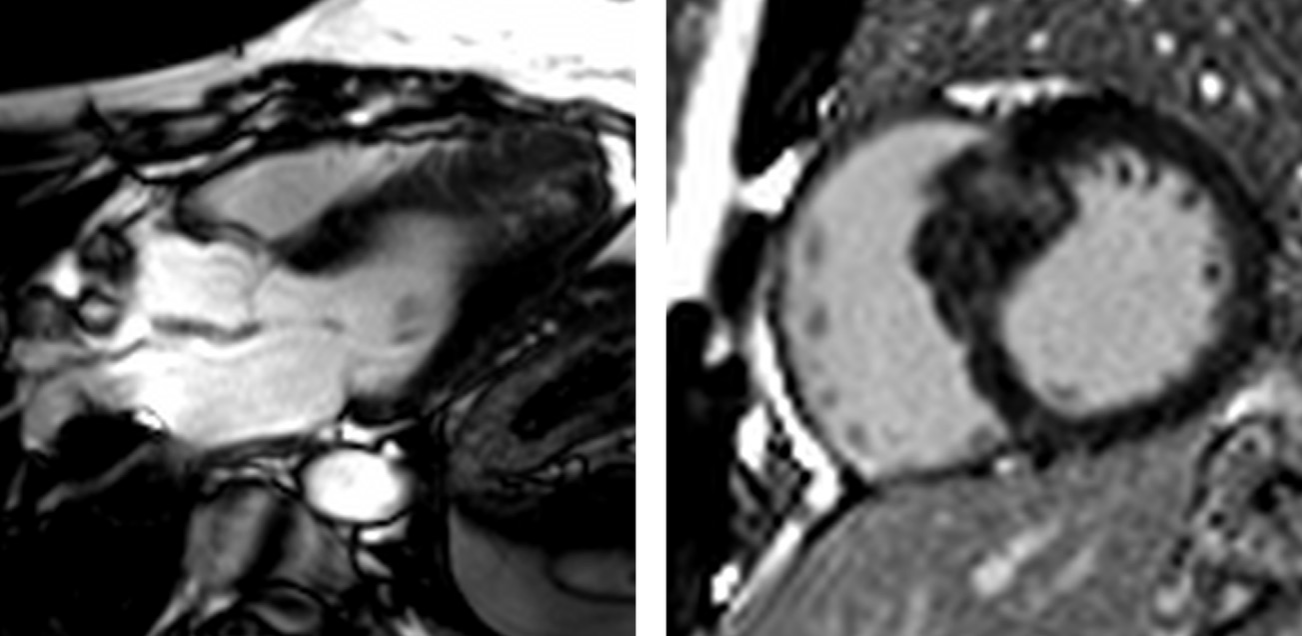
Hypertrophic cardiomyopathy examples of hypertrophic cardiomyopathy with typical septal hypertrophy (right) and an apical variant (left). Patchy non-infarct late gadolinium enhancement is seen within the regions of wall thickening.

Hypertrophic cardiomyopathy is the most common genetic cardiomyopathy. It is characterised by inappropriate regional wall thickening (figure 5), although there is significant phenotypic heterogeneity. Hypertrophic cardiomyopathy remains the leading cause of sudden cardiac death in young patients, although most patients have few clinical symptoms and overall event rates are low. As such, there is a major need for tools to improve patient risk stratification. Current risk stratification is imperfect, with strategies based on observational data.^36^

In addition to its diagnostic role, CMR has been proposed as a means of better identifying patients at high risk of sudden cardiac death. Although individual studies have been limited by small numbers, meta-analyses have demonstrated that both the presence and quantity of LGE are independently associated with all-cause and cardiac death.^37^ Importantly, the prevalence of LGE on CMR is around 50%–70%, ranging from patchy non-infarct distribution within hypertrophied segments to full-thickness enhancement with wall thinning in end-stage disease.^36^ Consequently, the positive predictive value of LGE for events remains low. However, the risk of sudden cardiac death appears to increase with increasing LGE burden (eg, HR 1.86 (95% CI 1.21 to 2.86) for 20% LGE; HR 3.45 (95% CI 1.46 to 8.16) for 40% LGE), suggesting that a minimum threshold for LGE volume may be of more clinical use. Further prospective studies are required to address this issue. As such, current 2014 European Society of Cardiology guidelines provide a class IIa (level of evidence B) recommendation for the assessment of myocardial fibrosis with CMR in hypertrophic cardiomyopathy, but do not integrate CMR into clinical risk stratification models.^36^

T1 mapping has been investigated in hypertrophic cardiomyopathy in small studies. Native T1 and ECV are able to differentiate healthy from diseased myocardium^21^ and may discriminate between hypertrophic cardiomyopathy and other conditions such as hypertensive heart disease.^38^ Beyond these preliminary data, however, the role of T1 mapping in hypertrophic cardiomyopathy requires further elucidation.

### Myocarditis

**Figure 6:**
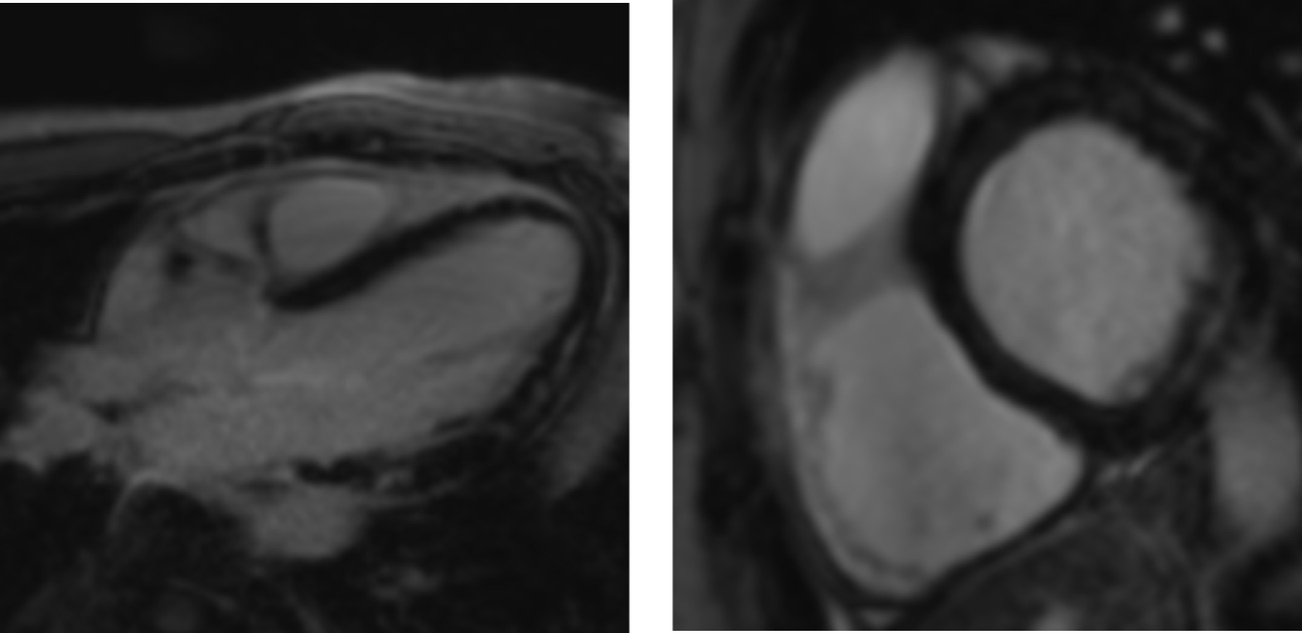
Myocarditis. Three-chamber (left) and short-axis (right) examples of patchy, non-infarct, mid-wall late gadolinium enhancement in the anterolateral and inferolateral walls of a patient with chronic myocarditis (3 months after the onset of symptoms). Note that these findings are non-specific.

Myocarditis is an inflammatory disease of the myocardium which can be acute, subacute or chronic and is caused by a variety of aetiologies—most commonly viral or idiopathic. The true incidence is difficult to ascertain but there appears to be a trend of increasing hospitalisation in recent years, in part due to high-sensitivity cardiac troponin assays and access to CMR.^39^ Myocarditis may be present in up to 12% of young adults presenting with sudden death and can lead to other diseases such as DCM.^40^ Adverse events in patients with confirmed myocarditis are lower in contemporary series than historic cohorts, but remain significant, particularly in patients with a complicated presentation (cardiac mortality and transplantation 11.3% at 1 year in patients with ejection fraction <50%, sustained ventricular arrhythmia or low output state).^41^ The initial inflammatory response, myocyte injury and tissue oedema may be followed by myocyte necrosis and the development of scar. Consequently, CMR is now an established imaging modality for the diagnosis of myocarditis. CMR uses T1-weighted and T2-weighted imaging, early gadolinium enhancement and LGE to detect hyperaemia, oedema and non-infarct replacement fibrosis, and diagnostic criteria (Lake Louise criteria) have been proposed based on observational data and expert consensus.^40 42^ LGE is seen in the majority of patients and is commonly distributed in the subepicardial inferior and lateral walls as well as the septum (figure 6).^43 44^ More recently, T1 mapping has been explored, with both native T1 and ECV demonstrating superior correlation with endomyocardial biopsy compared with Lake Louise criteria for the diagnosis of acute myocarditis (defined in this study as ≤14 days; area under the curve 0.77, 0.75 and 0.52, respectively).^45^ This highlights the ability of T1 mapping to detect increased extracellular volume due to oedema and reactive fibrosis during the early inflammatory response. Importantly, however, the diagnostic value of T1 mapping diminished in patients with symptoms >14 days.

The prognostic significance of CMR findings are restricted to LGE at present. The presence of non-infarct LGE is a powerful independent predictor of adverse events,^46^ even in patients without evidence of heart failure or left ventricular systolic dysfunction at presentation.^44^

### Infiltrative diseases

The most commonly encountered infiltrative cardiomyopathies are cardiac amyloidosis and sarcoidosis, which will be discussed here. Others include haemochromatosis and Fabry disease, both of which have characteristic findings on LGE, T1 and T2* mapping. These disease processes differ slightly from other pathologies as their hallmark is deposition of abnormal proteins within the interstitial space, rather than reactive inflammatory fibrosis and myocyte necrosis seen in previously discussed diseases. Research into these diseases is largely limited to observational studies.

**Figure 7:**
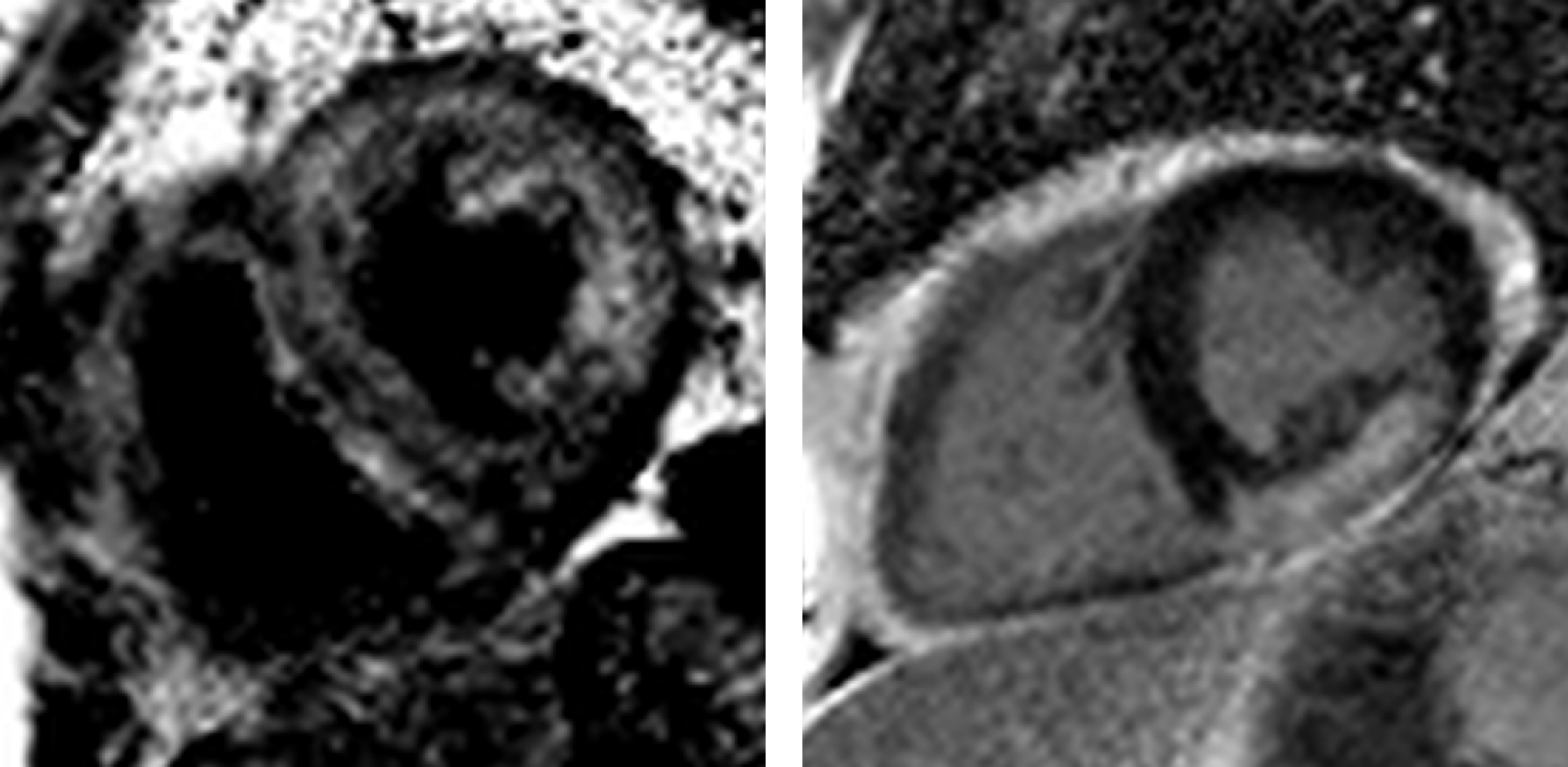
Cardiac amyloidosis and sarcoidosis. Left: cardiac amyloidosis. Note the black blood pool and diffuse late gadolinium enhancement within the abnormal myocardium. Right: cardiac sarcoidosis. The distribution of late gadolinium enhancement in cardiac sarcoidosis is variable. Here, there is a large burden of confluent enhancement in the inferior and inferolateral wall.

The hallmark of amyloidosis is extracellular deposition of fibrils, comprising low molecular weight subunits of serum proteins. The most common types of primary amyloidosis are light-chain and transthyretin (senile) cardiac amyloidosis. The former is a plasma cell dyscrasia which leads to monoclonal light-chain deposition, with cardiac involvement occurring in up to 50% of cases. This type of amyloidosis is responsible for the majority of systemic amyloidosis. The latter results in deposition of misfolded transthyretin and may be present in up to 10%–15% of older patients with heart failure. CMR offers an ideal modality for the diagnosis and assessment of cardiac amyloidosis given its ability to interrogate the cardiac interstitium. The presence of LGE is nearly universal in patients with confirmed cardiac amyloidosis.^47^ The typical distribution is global subendocardial or transmural LGE (figure 7), representing a gradient in burden of disease as assessed by ECV%.^48^ An additional typical finding in cardiac amyloid is difficulty in determining the optimal inversion time to null the myocardium. In other conditions, the blood pool is typically bright due to high concentrations of gadolinium; the null point of the blood pool is reached before the myocardium. In cardiac amyloidosis, the null point of the myocardium is reached before the blood pool due to high myocardial uptake and fast blood washout, resulting in a dark blood pool.^49^ CMR LGE in a characteristic pattern has been shown to have a high sensitivity and specificity for cardiac amyloidosis as well as prognostic power for mortality.^47–50^ ECV% and native T1 may also quantify amyloid burden and are independently associated with mortality.^51 52^ The incremental information provided by these CMR techniques is particularly pertinent given the recent successes of targeted therapies for transthyretin amyloid cardiomyopathy.

Sarcoidosis is a multiorgan, chronic, inflammatory granulomatous disease of unknown cause. Cardiac involvement occurs in up to one quarter of patients, although more than half of these patients may have subclinical disease.^53^ Classically the great mimic, cardiac sarcoidosis is frequently difficult. CMR is the diagnostic imaging modality of choice. Although there is no pathognomonic distribution of LGE (figure 7), typical patterns include multifocal LGE in a non-infarct pattern (although subendocardial disease is also seen) and direct extension of LGE across the septum from both right ventricular insertion points. The strength of CMR for the diagnosis of cardiac sarcoid lies largely in its sensitivity and excellent negative predictive value.^54^ As with other disease processes, the presence of LGE in cardiac sarcoidosis is of prognostic relevance, demonstrating an independent association with mortality.^55^ While the evidence base is somewhat limited, CMR LGE imaging is recommended in the current 2014 Heart Rhythm Society expert consensus criteria^56^ to aid in the diagnosis of cardiac sarcoidosis and as an arbiter of risk for ventricular arrhythmias—thus influencing decisions about invasive electrophysiology studies and primary prevention implantable cardioverter-defibrillators. Meanwhile, T1 and T2 mapping have been investigated as direct measures of inflammation, oedema and diffuse fibrosis, demonstrating an excellent ability to discriminate between patients with sarcoidosis and controls, outperforming current standard diagnostic criteria and additionally showing improvement after anti-inflammatory therapy.^57^

## Conclusion

Across a breadth of pathologies, myocardial fibrosis represents a final common pathway of myocardial disease, with the pattern and distribution of fibrosis differing between conditions. Irreversible replacement fibrosis, represented by LGE on CMR, is of nearly universal prognostic relevance. Meanwhile, diffuse interstitial fibrosis imaging has been shown to have great potential as a dynamic, early and reversible marker of myocardial disease. Both approaches are being used increasingly in clinical practice as diagnostic adjuncts, but data demonstrating that CMR improves outcomes in a cost-effective manner are lacking. Further clinical trials are required for this purpose, and several major observational and randomised controlled trials are currently underway.

**CME credits for Education in Heart**

Education in Heart articles are accredited for CME by various providers. To answer the accompanying multiple choice questions (MCQs) and obtain your credits, click on the ‘Take the Test’ link on the online version of the article. The MCQs are hosted on BMJ Learning. All users must complete a one-time registration on BMJ Learning and subsequently log in on every visit using their username and password to access modules and their CME record. Accreditation is only valid for 2 years from the date of publication. Printable CME certificates are available to users that achieve the minimum pass mark.

## References

[1] Treibel TA, López B, González A, et al Reappraising myocardial fibrosis in severe aortic stenosis: an invasive and non-invasive study in 133 patients. Eur Heart J 2018;39:699–709. 10.1093/eurheartj/ehx353 29020257 PMC5888951

[2] Treibel TA, Fontana M, Steeden JA, et al Automatic quantification of the myocardial extracellular volume by cardiac computed tomography: synthetic ECV by CCT. J Cardiovasc Comput Tomogr 2017;11:221–6. 10.1016/j.jcct.2017.02.006 28268091

[*3] Puntmann VO, Peker E, Chandrashekhar Y, et al T1 mapping in characterizing myocardial disease: a comprehensive review. Circ Res 2016;119:277–99. 10.1161/CIRCRESAHA.116.307974 27390332

[*4] Messroghli DR, Moon JC, Ferreira VM, et al Clinical recommendations for cardiovascular magnetic resonance mapping of T1, T2, T2* and extracellular volume: a consensus statement by the Society for (SCMR) endorsed by the European (EACVI). J Cardiovasc Magn Reson 2017;19 10.1186/s12968-017-0389-8 PMC5633041 28992817

[5] Chin CWL, Everett RJ, Kwiecinski J, et al Myocardial fibrosis and cardiac decompensation in aortic stenosis. JACC Cardiovasc Imaging 2017;10:1320–33. 10.1016/j.jcmg.2016.10.007 28017384 PMC5683736

[6] Treibel TA, Kozor R, Schofield R, et al Reverse myocardial remodeling following valve replacement in patients with aortic stenosis. J Am Coll Cardiol 2018;71:860–71. 10.1016/j.jacc.2017.12.035 29471937 PMC5821681

[7] Schulz-Menger J, Bluemke DA, Bremerich J, et al Standardized image interpretation and post processing in cardiovascular magnetic resonance: Society for Cardiovascular Magnetic Resonance (SCMR) board of trustees task force on standardized post processing. J Cardiovasc Magn Reson 2013;15:35 10.1186/1532-429X-15-35 23634753 PMC3695769

[8] Neumann F-J, Sousa-Uva M, Ahlsson A, et al 2018 ESC/EACTS guidelines on myocardial revascularization. Eur Heart J 2019;40:87–165. 10.1093/eurheartj/ehy394 30615155

[9] Kelle S, Roes SD, Klein C, et al Prognostic value of myocardial infarct size and contractile reserve using magnetic resonance imaging. J Am Coll Cardiol 2009;54:1770–7. 10.1016/j.jacc.2009.07.027 19874990

[10] El Aidi H, Adams A, Moons KGM, et al Cardiac magnetic resonance imaging findings and the risk of cardiovascular events in patients with recent myocardial infarction or suspected or known coronary artery disease. J Am Coll Cardiol 2014;63:1031–45. 10.1016/j.jacc.2013.11.048 24486280

[11] Ganesan AN, Gunton J, Nucifora G, et al Impact of late gadolinium enhancement on mortality, sudden death and major adverse cardiovascular events in ischemic and nonischemic cardiomyopathy: a systematic review and meta-analysis. Int J Cardiol 2018;254:230–7. 10.1016/j.ijcard.2017.10.094 29407096

[12] Dastidar AG, Baritussio A, De Garate E, et al Prognostic role of cardiac MRI and conventional risk factors in myocardial infarction with nonobstructed coronary arteries. JACC Cardiovasc Imaging 2019 10.1016/j.jcmg.2018.12.023. [Epub ahead of print: 13 Feb 2019]. 30772224

[13] Heitner JF, Senthilkumar A, Harrison JK, et al Identifying the infarct-related artery in patients with non-ST-segment-elevation myocardial infarction. Circ Cardiovasc Interv 2019;12:e007305 10.1161/CIRCINTERVENTIONS.118.007305 31035776

[14] Bulluck H, Hammond-Haley M, Fontana M, et al Quantification of both the area-at-risk and acute myocardial infarct size in ST-segment elevation myocardial infarction using T1-mapping. J Cardiovasc Magn Reson 2017;19:57 10.1186/s12968-017-0370-6 28764773 PMC5539889

[15] Liu D, Borlotti A, Viliani D, et al CMR native T1 mapping allows differentiation of reversible versus irreversible myocardial damage in ST-segment-elevation myocardial infarction: an OxAMI study (Oxford acute myocardial infarction). Circ Cardiovasc Imaging 2017;10:e005986 10.1161/CIRCIMAGING.116.005986 28798137 PMC5555391

[*16] Puntmann VO, Carr-White G, Jabbour A, et al Native T1 and ECV of noninfarcted myocardium and outcome in patients with coronary artery disease. J Am Coll Cardiol 2018;71:766–78. 10.1016/j.jacc.2017.12.020 29447739

[17] Treibel TA, Fridman Y, Bering P, et al Extracellular volume associates with outcomes more strongly than native or post-contrast myocardial T1. JACC: Cardiovascular Imaging 2019 10.1016/j.jcmg.2019.03.017 31103587

[18] Di Marco A, Anguera I, Schmitt M, et al Late gadolinium enhancement and the risk for ventricular arrhythmias or sudden death in dilated cardiomyopathy: systematic review and meta-analysis. JACC Heart Fail 2017;5:28–38. 10.1016/j.jchf.2016.09.017 28017348

[*19] Becker MAJ, Cornel JH, van de Ven PM, et al The prognostic value of late gadolinium-enhanced cardiac magnetic resonance imaging in nonischemic dilated cardiomyopathy: a review and meta-analysis. JACC Cardiovasc Imaging 2018;11:1274–84. 10.1016/j.jcmg.2018.03.006 29680351

[20] Iles L, Pfluger H, Phrommintikul A, et al Evaluation of diffuse myocardial fibrosis in heart failure with cardiac magnetic resonance contrast-enhanced T1 mapping. J Am Coll Cardiol 2008;52:1574–80. 10.1016/j.jacc.2008.06.049 19007595

[21] Puntmann VO, Voigt T, Chen Z, et al Native T1 mapping in differentiation of normal myocardium from diffuse disease in hypertrophic and dilated cardiomyopathy. JACC Cardiovasc Imaging 2013;6:475–84. 10.1016/j.jcmg.2012.08.019 23498674

[22] Puntmann VO, Carr-White G, Jabbour A, et al T1-Mapping and outcome in nonischemic cardiomyopathy: all-cause mortality and heart failure. JACC Cardiovasc Imaging 2016;9:40–50. 10.1016/j.jcmg.2015.12.001 26762873

[23] Køber L, Thune JJ, Nielsen JC, et al Defibrillator implantation in patients with nonischemic systolic heart failure. N Engl J Med 2016;375:1221–30. 10.1056/NEJMoa1608029 27571011

[24] Haugaa KH, Tilz R, Boveda S, et al Implantable cardioverter defibrillator use for primary prevention in ischaemic and non-ischaemic heart disease-indications in the post-DANISH trial era: results of the European Heart Rhythm Association survey. Europace 2017;19:660–4. 10.1093/europace/eux089 28431077

[25] Selvanayagam JB, Hartshorne T, Billot L, et al Cardiovascular magnetic resonance-guided management of mild to moderate left ventricular systolic dysfunction (CMR guide): study protocol for a randomized controlled trial. Ann Noninvasive Electrocardiol 2017;22 10.1111/anec.12420. PMC6931571 28117536

[26] Baumgartner H, Falk V, Bax JJ, et al 2017 ESC/EACTS guidelines for the management of valvular heart disease. Eur Heart J 2017;38:2739–91. 10.1093/eurheartj/ehx391 28886619

[*27] Bing R, Cavalcante JL, Everett RJ, et al Imaging and impact of myocardial fibrosis in aortic stenosis. JACC Cardiovasc Imaging 2019;12:283–96. 10.1016/j.jcmg.2018.11.026 30732723 PMC6361867

[28] Azevedo CF, Nigri M, Higuchi ML, et al Prognostic significance of myocardial fibrosis quantification by histopathology and magnetic resonance imaging in patients with severe aortic valve disease. J Am Coll Cardiol 2010;56:278–87. 10.1016/j.jacc.2009.12.074 20633819

[29] Dweck MR, Joshi S, Murigu T, et al Midwall fibrosis is an independent predictor of mortality in patients with aortic stenosis. J Am Coll Cardiol 2011;58:1271–9. 10.1016/j.jacc.2011.03.064 21903062

[30] Barone-Rochette G, Piérard S, De Meester de Ravenstein C, et al Prognostic significance of LGE by CMR in aortic stenosis patients undergoing valve replacement. J Am Coll Cardiol 2014;64:144–54. 10.1016/j.jacc.2014.02.612 25011718

[31] Musa TA, Treibel TA, Vassiliou VS, et al Myocardial scar and mortality in severe aortic stenosis. Circulation 2018;138:1935–47. 10.1161/CIRCULATIONAHA.117.032839 30002099 PMC6221382

[32] Everett RJ, Tastet L, Clavel M-A, et al Progression of hypertrophy and myocardial fibrosis in aortic stenosis: a multicenter cardiac magnetic resonance study. Circ Cardiovasc Imaging 2018;11:e007451 10.1161/CIRCIMAGING.117.007451 29914867 PMC6023592

[33] Bing R, Everett RJ, Tuck C, et al Rationale and design of the randomized, controlled Early Valve Replacement Guided by Biomarkers of Left Ventricular Decompensation in Asymptomatic Patients with Severe Aortic Stenosis (EVOLVED) trial. Am Heart J 2019;212:91–100. 10.1016/j.ahj.2019.02.018 30978556

[34] Lee H, Park J-B, Yoon YE, et al Noncontrast myocardial T1 mapping by cardiac magnetic resonance predicts outcome in patients with aortic stenosis. JACC Cardiovasc Imaging 2018;11:974–83. 10.1016/j.jcmg.2017.09.005 29153562

[35] Park S-J, Cho SW, Kim SM, et al Assessment of myocardial fibrosis using multimodality imaging in severe aortic stenosis: comparison with histologic fibrosis. JACC Cardiovasc Imaging 2019;12:109–19. 10.1016/j.jcmg.2018.05.028 30448148

[36] Elliott PM, Anastasakis A, Borger MA, et al 2014 ESC guidelines on diagnosis and management of hypertrophic cardiomyopathy: the task force for the diagnosis and management of hypertrophic cardiomyopathy of the European Society of Cardiology (ESC). Eur Heart J 2014;35:2733–79. 10.1093/eurheartj/ehu284 25173338

[37] Weng Z, Yao J, Chan RH, et al Prognostic value of LGE-CMR in HCM: a meta-analysis. JACC Cardiovasc Imaging 2016;9:1392–402. 10.1016/j.jcmg.2016.02.031 27450876

[38] Hinojar R, Varma N, Child N, et al T1 mapping in discrimination of hypertrophic phenotypes: hypertensive heart disease and hypertrophic cardiomyopathy: findings from the international T1 multicenter cardiovascular magnetic resonance study. Circ Cardiovasc Imaging 2015;8 10.1161/CIRCIMAGING.115.003285 26659373

[39] Shah Z, Mohammed M, Vuddanda V, et al National trends, gender, management, and outcomes of patients hospitalized for myocarditis. Am J Cardiol 2019;124:131–6. 10.1016/j.amjcard.2019.03.036 31060730

[40] Friedrich MG, Sechtem U, Schulz-Menger J, et al Cardiovascular magnetic resonance in myocarditis: a JACC white paper. J Am Coll Cardiol 2009;53:1475–87. 10.1016/j.jacc.2009.02.007 19389557 PMC2743893

[41] Ammirati E, Cipriani M, Moro C, et al Clinical presentation and outcome in a contemporary cohort of patients with acute myocarditis. Circulation 2018;138:1088–99. 10.1161/CIRCULATIONAHA.118.035319 29764898

[42] Caforio ALP, Pankuweit S, Arbustini E, et al Current state of knowledge on aetiology, diagnosis, management, and therapy of myocarditis: a position statement of the European Society of Cardiology Working Group on Myocardial and Pericardial Diseases. Eur Heart J 2013;34:2636–48. 10.1093/eurheartj/eht210 23824828

[43] Mahrholdt H, Goedecke C, Wagner A, et al Cardiovascular magnetic resonance assessment of human myocarditis: a comparison to histology and molecular pathology. Circulation 2004;109:1250–8. 10.1161/01.CIR.0000118493.13323.81 14993139

[44] Aquaro GD, Perfetti M, Camastra G, et al Cardiac MR with late gadolinium enhancement in acute myocarditis with preserved systolic function: ITAMY study. J Am Coll Cardiol 2017;70:1977–87. 10.1016/j.jacc.2017.08.044 29025554

[45] Lurz P, Luecke C, Eitel I, et al Comprehensive cardiac magnetic resonance imaging in patients with suspected myocarditis: the MyoRacer-Trial. J Am Coll Cardiol 2016;67:1800–11. 10.1016/j.jacc.2016.02.013 27081020

[*46] Gräni C, Eichhorn C, Bière L, et al Prognostic value of cardiac magnetic resonance tissue characterization in risk stratifying patients with suspected myocarditis. J Am Coll Cardiol 2017;70:1964–76. 10.1016/j.jacc.2017.08.050 29025553 PMC6506846

[47] Syed IS, Glockner JF, Feng D, et al Role of cardiac magnetic resonance imaging in the detection of cardiac amyloidosis. JACC Cardiovasc Imaging 2010;3:155–64. 10.1016/j.jcmg.2009.09.023 20159642

[48] Fontana M, Pica S, Reant P, et al Prognostic value of late gadolinium enhancement cardiovascular magnetic resonance in cardiac amyloidosis. Circulation 2015;132:1570–9. 10.1161/CIRCULATIONAHA.115.016567 26362631 PMC4606985

[49] Maceira AM, Joshi J, Prasad SK, et al Cardiovascular magnetic resonance in cardiac amyloidosis. Circulation 2005;111:186–93. 10.1161/01.CIR.0000152819.97857.9D 15630027

[50] Raina S, Lensing SY, Nairooz RS, et al Prognostic value of late gadolinium enhancement CMR in systemic amyloidosis. JACC Cardiovasc Imaging 2016;9:1267–77. 10.1016/j.jcmg.2016.01.036 27568115

[51] Banypersad SM, Fontana M, Maestrini V, et al T1 mapping and survival in systemic light-chain amyloidosis. Eur Heart J 2015;36:244–51. 10.1093/eurheartj/ehu444 25411195 PMC4301598

[52] Knight DS, Zumbo G, Barcella W, et al Cardiac structural and functional consequences of amyloid deposition by cardiac magnetic resonance and echocardiography and their prognostic roles. JACC: Cardiovascular Imaging 2019;12:823–33. 10.1016/j.jcmg.2018.02.016 29680336

[53] Hamzeh N, Steckman DA, Sauer WH, et al Pathophysiology and clinical management of cardiac sarcoidosis. Nat Rev Cardiol 2015;12:278–88. 10.1038/nrcardio.2015.22 25707386

[54] Smedema J-P, Snoep G, van Kroonenburgh MPG, et al Evaluation of the accuracy of gadolinium-enhanced cardiovascular magnetic resonance in the diagnosis of cardiac sarcoidosis. J Am Coll Cardiol 2005;45:1683–90. 10.1016/j.jacc.2005.01.047 15893188

[55] Greulich S, Deluigi CC, Gloekler S, et al CMR imaging predicts death and other adverse events in suspected cardiac sarcoidosis. JACC Cardiovasc Imaging 2013;6:501–11. 10.1016/j.jcmg.2012.10.021 23498675

[56] Birnie DH, Sauer WH, Bogun F, et al HRS expert consensus statement on the diagnosis and management of arrhythmias associated with cardiac sarcoidosis. Heart Rhythm 2014;11:1304–23. 10.1016/j.hrthm.2014.03.043 24819193

[57] Puntmann VO, Isted A, Hinojar R, et al T1 and T2 mapping in recognition of early cardiac involvement in systemic sarcoidosis. Radiology 2017;285:63–72. 10.1148/radiol.2017162732 28448233

[58] Everett RJ, Stirrat CG, Semple SIR, et al Assessment of myocardial fibrosis with T1 mapping MRI. Clin Radiol 2016;71:768–78. 10.1016/j.crad.2016.02.013 27005015

